# Advances in autonomic dysfunction research in Parkinson’s disease

**DOI:** 10.3389/fnagi.2025.1468895

**Published:** 2025-03-12

**Authors:** Hongjia Xu, Xiaolei Zheng, Xinyue Xing, Zhichao Bi, Dewei Wang, Cheng Zhang, Lifei Wei, Yulin Jin, Shunliang Xu

**Affiliations:** ^1^Department of Neurology, The Second Hospital, Cheeloo College of Medicine, Shandong University, Jinan, China; ^2^Department of Human Genetics, School of Medicine, Emory University, Atlanta, GA, United States

**Keywords:** Parkinson’s disease, autonomic dysfunction, review, motor symptoms, non-motor symptoms

## Abstract

Parkinson’s disease (PD) is a prevalent neurodegenerative disorder, best known for its motor symptoms such as resting tremor, muscle rigidity, and bradykinesia. However, autonomic dysfunction is an important non-motor aspect that often brings considerable discomfort and distress to both patients and their families. In this review, we summarize recent advances in understanding the pathophysiological mechanisms of autonomic dysfunction and explore its relationship with other clinical features. Our aim is to discover novel potential diagnostic and therapeutic strategies, alleviate patient suffering, and pave the way for future clinical and basic research.

## 1 Introduction

Parkinson’s disease (PD) is a common and complex neurodegenerative disease. While PD is most typically observed in the elderly, a growing hypothesis suggests that a prodromal phase, marked by a range of early symptoms, may begin as early as a person’s 20s (Darweesh et al., 2018; Fereshtehnejad et al., 2019). Studied for centuries, the hallmark pathophysiology of PD is involves by a decrease in dopamine in substantia nigra neurons and striatum, and the formation of intracellular inclusion bodies that contain α-synuclein(α-syn) aggregates (Shahmoradian et al., 2019; Tolosa et al., 2021; Chu et al., 2024). The clinical features include motor symptoms represented by bradykinesia, resting tremor, increased muscle tone, and gait abnormalities, as well as non-motor symptoms such as olfactory disturbances, sleep disturbances, cognitive dysfunction, emotional abnormalities, and autonomic dysfunction (Bloem et al., 2021). Autonomic dysfunction (AutD) is an important non-motor symptoms in PD, which includes gastrointestinal dysfunction, cardiovascular dysfunction, urinary dysfunction, thermoregulatory dysfunction, pupillary motor function and sexual dysfunction (Chen et al., 2020; Pfeiffer, 2020). It has been reported that 70–80 per cent of patients may suffer from gastrointestinal autonomic dysfunction (Perez-Pardo et al., 2017). Additionally, about 30–50 per cent of patients experience orthostatic hypotension. Cardiovascular dysfunctions significantly impact patients’ lives by causing extreme blood pressure instability, which not only affects the cognitive functioning but also weakens their ability to perform daily activities (Palma and Cortelli, 2023; Palma et al., 2024). Furthermore, slow gastric emptying and constipation can impair the pharmacodynamics of medications, leading to a deterioration of motor function (Chung and Pfeiffer, 2020; Leta et al., 2023).

Research has revealed that AutD may appear years before than motor symptoms in Parkinson’s disease, and more than half of PD patients experience at least one form of the AutD, which can significantly impact their quality of life (Bloem et al., 2021; Longardner et al., 2022). Moreover, AutD is considered a key feature in the diagnosis of prodromal PD and serves a prognostic biomarker (Schapira et al., 2017; Blesa et al., 2021). Although the current understanding of AutD in PD is well documented, its precise pathogenesis and relationship to other PD symptoms are remain unclear (Chaudhuri, 2021).

Given the significant impact of AutD on the lives of PD patients, and with the International Parkinson and Movement Disorder Society (MDS) introducing various clinical rating scales to assess it, AutD has become a cutting-edge area of PD research (Chen et al., 2020; Chaudhuri, 2021). This review highlights recent understanding of AutD in PD. In contrast to previous reviews, we summarize current research on the correlation between autonomic dysfunction and other related clinical symptoms in patients with PD. Our review describes some of the common pathogenetic mechanisms that have been identified, thus filling the gaps in this area, aiming to explore more diagnostic and therapeutic approaches to autonomic dysfunction and lay the foundation for future high-quality clinical and basic research.

## 2 Pathogenesis of autonomic dysfunction in PD

Dopaminergic deficits in nigral neurons and the striatum, along with the accumulation of α-synuclein in intraneuronal inclusion bodies, are recognized as characteristic neuropathological features of Parkinson’s disease (PD). In addition to these well-known mechanisms, other theories suggest that PD pathogenesis may also involve factors such as traumatic injury, genetic mutations, mitochondrial dysfunction, and neuroinflammation. However, the exact pathogenesis of autonomic disorders in PD has not yet been clearly demonstrated. Current research suggests multiple factors may contribute to the pathogenesis of AutD in PD, potentially involving abnormal protein deposition and neuronal destruction, genetic problems, and environmental factors (Chen et al., 2020; Bloem et al., 2021).

### 2.1 Abnormal protein deposition and neuronal destruction

The deposition of alpha-synuclein(α-syn) and phosphorylated α-syn in autonomic neurons and their innervated regions, along with the neuronal degeneration, are considered as key factors contributing to autonomic dysfunction in patients with Parkinson’s disease (PD). PD with autonomic impairment is recognized to involve varying degrees of α-syn accumulation and neuronal destruction across both central and peripheral nerves (Pfeiffer, 2020). For example, in the hypothalamus of PD patients with autonomic dysfunction, α-syn deposition was found in the paraventricular, infundibular, and supraoptic nuclei, but the severity of symptoms was not found to be significantly correlated with alpha-syn deposition (De Pablo-Fernandez et al., 2017). Additionally, α-syn deposition has been detected in other central autonomic control centres, such as those in the cortex, brainstem, and spinal cord, which are hypothesized to contribute to autonomic dysfunction in PD (Christopher et al., 2014; De Pablo-Fernandez et al., 2017; Chen et al., 2020).

Neurosonography of PD patients with parasympathetic dysfunction revealed that these patients exhibited a smaller vagal cross-sectional area compared to controls. This reduction was considered to be caused by α-syn deposition, leading to progressive neurodegenerative lesion (Huckemann et al., 2023). Studies in 1-methyl-4-phenyl-1,2,3,6-tetrahydropyridine(MPTP)-injected mice have shown that phosphorylated α-syn can accumulate in Schwann cells within the vagus nerve, where it interacts with Toll-like receptors to induce apoptosis in these cells. This process is thought to lead to autonomic dysfunction in mice, manifesting primarily as impairments in the gastrointestinal, cardiovascular, and urinary systems (Li et al., 2024). In transgenic mouse model of PD, α-syn deposits were observed to accumulate with age in the colon’s submucosal and myenteric plexus neurons, leading to autonomic dysfunction, predominantly manifesting as constipation. Notably, this pathological change in the gut appeared before the onset of motor symptoms, suggesting that α-syn may spread from the gut to the brain via the vagus nerve (Chen et al., 2018). This finding may explain why some PD patients initially develop autonomic deficits in the gastrointestinal tract. However, a subsequent study found that α-syn deposition in the gastrointestinal tract did not directly affect the autonomic function of its associated segments, suggesting that α-syn may not be the primary driver of gastrointestinal autonomic dysfunction (Lee et al., 2018). Further studies are needed to determine whether gastrointestinal autonomic dysfunction is indeed related to α-syn aggregation.

Research on sympathetic nerves has yet to fully explain the mechanism underlying norepinephrine deficiency in some PD patients. A team has proposed a novel mechanism, which find that α-syn deposits are present in sympathetic nerves within blood vessels, sweat glands, erector spinae, and myocardium. This mechanism suggests that α-syn deposits in sympathetic nerves may contribute to autonomic dysfunction in PD, particularly cardiovascular and thermoregulatory issues, by disrupting norepinephrine release through sympathetic nerve inactivation. However, further studies are required to confirm this hypothesis (Isonaka et al., 2019).

### 2.2 Genetic factors

Parkinson’s disease is also a hereditary disorder, with a recent review in The Lancet indicating that the genetic risk of Parkinson’s disease ranges from 22 to 40% (Ben-Shlomo et al., 2024; Morris et al., 2024). Of these, about 10% are due to single-gene mutations, with common mutations occurring in genes like Leucine-rich repeat kinase 2 (LRRK2), Parkin RBR E3 Ubiquitin Protein Ligase (PRKN), and Synuclein Alpha (SNCA) (Wanneveich et al., 2018; Isonaka et al., 2021). In addition, genes such as mutations in the β-glucoside cerebroside gene (GBA) have also been proposed, with patients carrying these mutations exhibiting more severe motor symptoms and greater likelihood of autonomic dysfunction in Parkinson’s disease (Gonçalves et al., 2021).

**FIGURE 1:**
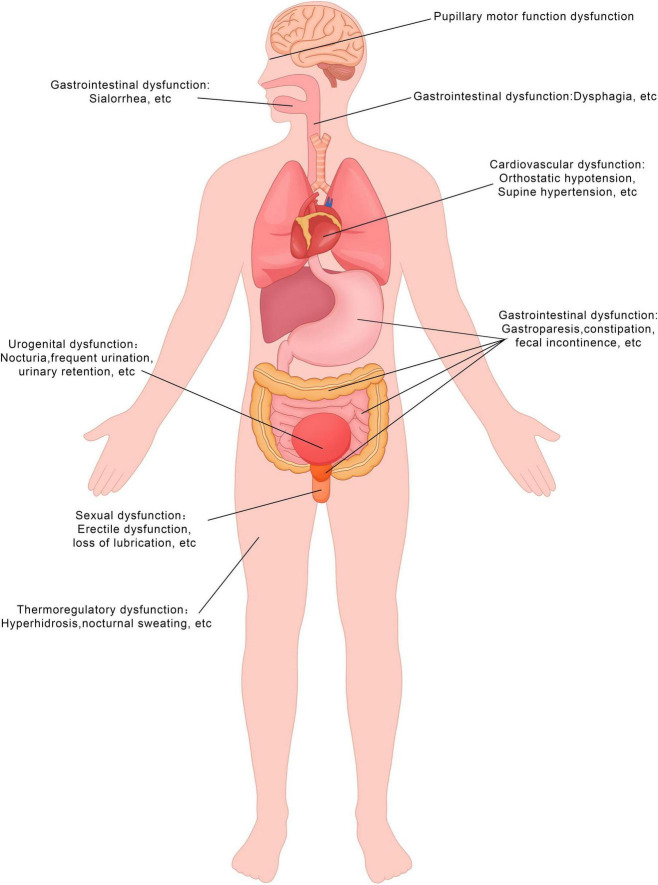
Symptoms of autonomic dysfunction in the PD patients. This figure demonstrates the types of symptoms experienced by patients. The diagram was produced and generated by Adobe Photoshop.

The SNCA gene was the first gene identified as the causative factor of PD. Different point mutations in this gene are associate with different clinical manifestations, with the more recurrent mutations, more likely to be linked to prominent non-motor symptoms, including autonomic dysfunction (Magistrelli et al., 2021). PD patients with E46K-SNCA mutation experience prominent non-motor symptoms. A comparison of skin biopsies from E46K mutation in alpha-synuclein gene (E46K-SNCA) carriers, parkin gene mutation (PARK2) carriers, and healthy controls revealed that E46K-SNCA carriers exhibited more substantial α-syn deposits and a marked reduction in small nerve fiber density. Previous studies have shown pronounced denervation of cardiovascular autonomic nerves in these carriers, supporting the view that E46K-SNCA mutations are associated with significant autonomic dysfunction compared to other genotypes (Carmona-Abellan et al., 2019). Two other common variants, A30P and A53T, also show autonomic dysfunction, significant impairment of the gastrointestinal dysfunction is observed in animal studies (Carmona-Abellan et al., 2019). In addition, rs11931074 mutation in the SNCA gene has been linked to α-syn deposition in the enteric nervous system of PD patients, which impacts gastrointestinal autonomic dysfunction in PD patients (Chung et al., 2019). In contrast, PD patients with the G2385R mutation in LRRK2 exhibit higher prevalence and severity of autonomic dysfunction-primarily in cardiovascular, gastrointestinal, and urinary functions, but the specific pathogenesis of this needs to be further investigated (Yang et al., 2021).

**TABLE 1:** The treatments for autonomic dysfunction in PD patients.

Treatment	Mechanism	Type of AutD	Possible adverse reactions	References
**Traditional**				
Droxidopa	Norepinephrine prodrug	Cardiovascular dysfunction	Hypertension, headache	Hauser et al., 2014a; Hauser et al., 2014b
Fludrocortisone	Mineralocorticoid	Cardiovascular dysfunction	Hypertension, hypokalemia	Schoffer et al., 2007; Schreglmann et al., 2017
Midodrine	Alpha-1 agonist	Cardiovascular dysfunction	Hypertension	Joseph et al., 1993; Smith et al., 2016
Domperidone	D2 antagonist	Cardiovascular dysfunction	QT prolongation, ventricular tachyarrhythmia	Schoffer et al., 2007
Polyethylene glycol	Osmotic laxative	Gastrointestinal dysfunction	Nausea, diarrhea	Zangaglia et al., 2007
Lubiprostone	Intestinal activator of chloride channel type 2	Gastrointestinal dysfunction	Nausea, diarrhea	Ondo et al., 2012
Probiotic strains	Intestinal microbiota modulation	Gastrointestinal dysfunction		Barichella et al., 2016; Leta et al., 2021b
Prebiotic fibers	Alters stools consistency	Gastrointestinal dysfunction		Barichella et al., 2016
Solifenacin	Muscarinic (M3) inhibitor	Urinary dysfunction	Xerostomia, constipation, dyspepsia, blurred vision	Zesiewicz et al., 2015
Sildenafil	Inhibitor of the phosphodiesterase type 5	Sexual dysfunction	Headache, hypotension, dyspepsia	Bernard et al., 2016
**New**				
Fipamezole	Alpha-2 agonist	Cardiovascular dysfunction	Hypertension	Leta et al., 2019; Rukavina et al., 2022
Atomoxetine	Norepinephrine transporter blocker	Cardiovascular dysfunction	Liver damage	Shibao et al., 2007; Ramirez et al., 2014; Palma and Kaufmann, 2018
Pyridostigmine	Acetylcholinesterase inhibitor	Cardiovascular dysfunction	Nausea, diarrhea	Byun et al., 2017
Linaclotide	Guanylate cyclase C agonist	Gastrointestinal dysfunction	Abdominal pain, diarrhea	Freitas et al., 2018
Prucalopride	5-HT4 agonist	Gastrointestinal dysfunction	Nausea, diarrhea, headache	Freitas et al., 2018
Elobixibat	Ileal bile acid transporter inhibitor	Gastrointestinal dysfunction	Abdominal pain, diarrhea	Nakajima et al., 2018
Mirabregon	β-3 adrenoreceptor agonist	Urinary dysfunction	Urinary tract infection, tachycardia	Peyronnet et al., 2018; Cho et al., 2021
Botulin toxin A	Inhibition of acetylcholine release	Urinary dysfunction	Urinary tract infection, postvoid residual	Kulaksizoglu and Parman, 2010; Giannantoni et al., 2011

Heart rate and blood pressure are among the earliest and most common indicators of cardiovascular dysfunction in PD patients. Analysis of PD patients using heart rate variability (HRV) has shown that those with GBA mutations tend to have higher resting and upright heart rates, along with greater blood pressure decreases upon standing, compared to patients with idiopathic PD and healthy controls, suggesting parasympathetic impairment. Carandina et al. (2022) propose that the GBA mutation may lead to a deficiency in the lysosomal enzyme glucocerebrosidase (GCase), which impairs the breakdown of lysosomal α-synuclein, resulting in significant cardiovascular autonomic dysfunction. A similar pathogenic process may also underlie gastrointestinal autonomic dysfunction, particularly constipation, in PD patients with GBA mutations. Further analysis has indicated that PD patients with GBA mutations experience impaired sweat gland function, primarily in the distal limbs, with skin biopsies revealing severe autonomic nerve loss and reduced sweat gland density due to α-synuclein deposition (Carandina et al., 2022; Devigili et al., 2023). Recently, Giulia’s team concluded that while patients with GBA mutations are more likely to develop orthostatic hypotension (OH), its severity did not appear to be significantly different between GBA-PD and non-GBA-PD patients after instrumental assessment. This finding suggests that further studies are needed to explore the underlying mechanisms (Giannini et al., 2024).

### 2.3 Other possible factors

A recent research has proposed that abnormal functional connectivity between the insula and limbic lobes in patients with early-stage Parkinson’s disease may contribute to severe autonomic dysfunction symptoms (Conti et al., 2024). In addition, secretion, transport and release of central transmitters play crucial roles in autonomic dysfunction observed in PD patients. In particular, Dopamine is essential in regulating autonomic functions. A study of cranial brain imaging and autonomic dysfunction in 310 PD patients found that degeneration of dopaminergic neurons in the striatum is linked to autonomic dysfunction, primarily affecting gastrointestinal and cardiovascular functions (van Deursen et al., 2020). Urinary tract disorders in PD are believed to arise from multiple factors. Given that most PD patients are elderly, it is essential to determine whether urinary symptoms in male patients are due to prostate enlargement or PD-related urinary dysfunction (Valentino et al., 2018). The severity of striatal dopamine transporter deficiency has been correlated with bladder symptoms (Metzger and Emborg, 2019). Additionally, PD pathology affects other anatomical structures involved in bladder function, including the raphe nuclei and the locus coeruleus (VanderHorst et al., 2015). Abnormal pupillary function in PD may also be linked to the loss of dopamine in the central nervous system and retina, which in turn leads to visual hallucinations and ocular dyskinesia. In cases of pupillary constriction disorders, neuron loss in the Edinger-Westphal nucleus is implicated in the pathogenesis of PD (Metzger and Emborg, 2019).

The gut-brain axis has emerged as a hotspot for research into the pathogenesis of autonomic dysfunction in PD. The digestive tract is the main location where interactions occur between the outside environment and the body’s internal environment. Environmental changes in the gastrointestinal tract, especially changes in gastrointestinal flora have been found to have an important role in the pathogenesis of PD. Many studies have found that changes in gastrointestinal flora are correlate with cognitive and motor deficits in PD patients (Barichella et al., 2018; Matheoud et al., 2019; Pietrucci et al., 2019; Chen et al., 2020). Although some studies have pointed out that changes in gastrointestinal flora could trigger gastrointestinal inflammatory, autoimmune reactions, and are associated with α-syn deposition, which can spread to the brain and other nervous systems via the vagus nerve. However, no direct correlation has been found between these changes and gastrointestinal dysfunction or other autonomic dysfunction in patients in either basic experiments or clinical studies (Claudino dos Santos et al., 2023). There have been studies that have found bile acid abnormalities in PD patients with abnormal lipid metabolism, which are caused by an imbalance in the intestinal flora. Therefore, PD patients may also be suffering from an imbalance of intestinal flora that affects the biochemical metabolism of the gastrointestinal tract and thus indirectly affects the autonomic function of the gastrointestinal tract (Hasuike et al., 2020; Claudino dos Santos et al., 2023; Higinbotham and Kilbane, 2024).

## 3 Relationship of autonomic dysfunction to other clinical symptoms of PD

### 3.1 Relationship to motor symptoms

Neurodegenerative lesions occur in various brainstem regions of PD patients, including the substantia nigra and locus coeruleus. Most of these affected areas were involved in both motor postural control and autonomic regulation (Benarroch, 1993; Seidel et al., 2014), suggest a potential direct link between motor symptoms and autonomic dysfunction.

PD patients with multiregional damage exhibit significant slowness of movement, lower oxygen pulse, oxygen consumption, systolic blood pressure, and respiratory exchange ratio (RER) at maximal exercise load during the 10-meter walk test. These observation suggested that more severe autonomic dysfunction is associated with poorer exercise capacity (Qin et al., 2024). Additional studies have confirmed higher Scales for Outcomes in Parkinson’s disease - Autonomic (SCOPA-AUT) scores in patients with indeterminate subtypes of PD compared with patients with tremor dominant (TD) and postural instability gait disorder (PIGD) subtypes, suggesting that there may be differences in the severity and progression of autonomic dysfunction across PD subtypes (Jeong et al., 2021). However, the relationship between exercise capacity and autonomic dysfunction in patients with PD is not fully understood and further studies are needed to clarify it. One hypothesis suggests that the co-occurrence of both conditions may be attributed to the presence of Lewy bodies, which are extensively distributed in the hypothalamus, the lateral reticular nucleus of the medulla oblongata, sympathetic ganglia, the dorsal nucleus of the vagus nerve, and sacral parasympathetic nuclei within the spinal cord. This distribution may disrupt autonomic regulatory mechanisms and diminish maximum sympathetic activation during movement, thereby impacting patient mobility (Qin et al., 2024).

Some studies found a strong association between autonomic dysfunction (e.g., OH, gastrointestinal symptoms, etc.) and gait disturbance and falls in patients with advanced PD. Kwon et al. (2021a) proposed that early gastrointestinal and axial symptoms in PD may be interconnected within the pathophysiology of the condition. However, the precise mechanisms remain unclear and necessitate further detailed investigation (Kwon et al., 2021a). PIGD has been found to be significantly and positively correlated with the SCOPA-AUT total score and the score of urinary symptoms in patients with PD. Autonomic dysfunction in these patients can impact gait, particularly in the early to mid-stages of the disease (Kwon et al., 2021b; Zhou et al., 2023). The more severe autonomic dysfunction in patients with new-onset PD is associated with poorer performance in gait speed, stride length, walking rhythm, and more pronounced difficulties with backward movement. Notably, urinary autonomic abnormalities in new-onset PD patients are strongly correlated with gait impairment. The research team posits that severe autonomic dysfunction may signify more extensive brain damage, including regions such as the pontine urination center and the periaqueductal gray matter of the midbrain, thereby indicating a potential for comorbidity (Lee et al., 2023).

Postural instability was first linked to autonomic dysfunction in a study by You and colleagues, who found that postural instability in PD patients was associated with parasympathetic autonomic dysfunction (You et al., 2020). Another prospective study that followed 50 PD patients demonstrated that those with cardiovascular dysautonomia (including but not limited to orthostatic hypotension) were more likely to fall. PD patients with more cardiac sympathetic modulation required more efforts to maintain balance in standing (Romagnolo et al., 2018). In 2020, two research teams led by You and Yoan posits that the autonomic and postural pathways share critical relay points within the brainstem, cerebral cortex, and basal ganglia. Autonomic dysfunction resulting from a loss of dominance in cardiac sympathetic innervation and impairment of parasympathetic nerves contributes to alterations in postural control due to disrupted communication between the cerebral cortex and brainstem (Espinoza-Valdés et al., 2020; You et al., 2020). It can be argued that cardiovascular autonomic deficits may be a strong, independent predictor of falls in patients with PD. Therefore, clinicians should be aware of the possibility of postural instability associated with autonomic dysfunction, even though the patient does not have typical postural instability.

### 3.2 Relationship to non-motor symptoms

In addition to motor symptoms, cognitive impairment, sleep disorders and emotions changes have been found to strongly correlated with autonomic dysfunction in PD. These symptoms significantly affect the life quality of PD patients and have become a hot topic of research in recent years.

#### 3.2.1 Relationship to cognitive impairment

Numerous studies have shown that PD patients with autonomic dysfunction are more likely to experience cognitive impairment. In fact, PD patients may exhibit autonomic symptoms and subtle cognition changes several years before a formal diagnosis, which may affect their differential diagnosis from other α-synucleinopathies (Palermo et al., 2020). Magdalena and coworkers reviewed several studies and found that approximately 25% of these patients showed mild cognitive impairment (MCI) at an early stage, with most also showing blood pressure abnormalities, such as orthostatic hypotension (OH). This may be due to frequent cranial hypoperfusion caused by cardiovascular autonomic dysfunction, leading to unstable blood pressure or neurodegenerative disease affecting central or peripheral noradrenergic pathways (McDonald et al., 2016). However, Magdalena noted that it remains unclear whether the relationship between the cardiovascular system and cognitive impairment is causal or simply correlative, highlighting the need for more rigorous controlled trials to clarify this link (Kwaśniak-Butowska et al., 2021). Recently, Ruiz-Barrio et al. (2023) performed a retrospective analysis and identified that early-stage orthostatic hypotension (OH) is linked to an elevated risk of cognitive impairment. They elucidated that the detrimental effects of OH on cognitive function arise from recurrent episodes of cerebral hypoperfusion, which induce chronic hypoxic changes, thereby activating specific molecular pathways that lead to non-specific neuronal destruction and neurodegeneration. Additionally, they suggested the potential for treating OH as a means to prevent cognitive decline (Ruiz-Barrio et al., 2023).

It has been reported that mild cognitive impairment in patients with new-onset PD is often associated with gastrointestinal symptoms related to autonomic dysfunction, particularly memory and executive function deficits (Jones et al., 2020). One study found that more severe gastrointestinal symptoms predicted a trend toward declining performance on alphabetic fluency, visuospatial, learning, and memory in patients with up to 5-year follow-up period. Notably, these cognitive declines were linked specifically to gastrointestinal autonomic symptoms, rather than to non-autonomic symptoms, suggesting that gastrointestinal symptoms may serve as a predictive marker of cognitive decline in PD patients (Jones et al., 2020). By studying early Parkinson’s patients, a team found that degeneration of the LocusCoeruleus leads to the onset of cognitive deficits and worsening of gastrointestinal symptoms at subsequent follow-up (Kim et al., 2024). Additionally, Camacho and colleagues conducted a cohort study revealing that PD patients exhibiting early symptoms of constipation are at an increased risk of developing dementia. Furthermore, the severity of constipation at disease onset serves as a prognostic indicator for accelerated dementia progression (Camacho et al., 2021). However, the precise mechanisms and causal relationship between cognitive impairment and gastrointestinal symptoms remain unclear. The leading hypothesis suggests that changes in gut microbiology may influence cognitive dysfunction in patients with PD. In the early stages of certain Parkinson’s disease (PD) patients, aggregates of alpha-synuclein protein are observed to accumulate in the gastrointestinal tract. Metabolites resulting from intestinal dysregulation may lead to increased gut permeability, oxidative stress, and localized inflammation, which can influence cerebral function via the gut-brain axis. This cascade ultimately results in damage and deposition of alpha-synuclein, contributing to neurodegeneration within the brain and subsequent cognitive impairment (Nair et al., 2018; Dowling et al., 2022; Warnecke et al., 2022). Additionally, degenerative changes in both the peripheral gastrointestinal system and the central cholinergic system may play a role, potentially impacting both gastrointestinal symptoms and cognitive function. In patients with autonomic dysfunction, early degeneration of cholinergic neurons within the gastrointestinal tract can be observed, contributing to the manifestation of autonomic impairment. Furthermore, cholinergic neurons in the brain primarily function as projection neurons connecting various central nervous system (CNS) regions. Along with motor neurons and certain autonomic neurons, these neurons facilitate interactions between the CNS and peripheral nervous system. The concurrent degeneration of these neuronal populations may adversely impact cognitive functions in Parkinson’s disease (PD) patients with autonomic dysfunction (Titova et al., 2016; Bohnen et al., 2022). Therefore, if PD patients suffer from autonomic dysfunction, clinicians should assess for the signs of mild cognitive impairment (Kwon et al., 2022).

#### 3.2.2 Relationship to sleep disorders

Rapid eye movement sleep behavior disorder (RBD) is an important non-motor symptom of PD. Patients with RBD tend to experience more severe motor and non-motor symptoms, with RBD categorized as either isolated or secondary. The isolated RBD is considered a prodromal symptom of PD as its high rate of progression to the disease (Toft et al., 2021). Sleep disturbances and autonomic dysfunction are both key non-motor symptoms that substantially impact the quality of life of PD patients. Several studies have demonstrated a correlation between RBD and autonomic dysfunction in PD, showing that PD patients with RBD have more severe involuntary dysfunction than those without RBD. Furthermore, the severity of autonomic symptoms may be linked to a faster phenotypic progression in patients with isolated RBD (Kim et al., 2016; Li et al., 2017).

To clarify which autonomic symptoms are associated with RBD in PD, Fujita et al. (2022) conducted a followed up study on 126 PD patients with RBD. They found that the cardiovascular and urinary symptoms were the most severe among autonomic symptoms, with urinary symptoms-particularly “weak urinary stream”-emerging as a key indicator for worsening RBD. This finding suggests that the disease-specific pathology in brainstem nuclei, such as the periaqueductal gray (PAG) and pontine micturition center (PMC), may be more pronounced in PD patients with RBD (Fujita et al., 2022). Kim et al. (2016) reported a strong association between early-stage RBD with OH and cardiac sympathetic denervation, suggesting that cardiovascular symptoms in RBD may be linked to sympathetic denervation.

Another hypothesis suggested that autonomic dysfunction and sleep disorders, particularly RBD, may share overlapping areas of co-morbidity (Cortellia and Lambardi, 2005). A longitudinal cohort study found that all autonomic symptoms-except for pupillary movement-were more severe and deteriorated more rapidly in PD patients with RBD compared to those without RBD (Ashraf-ganjouei et al., 2021; Maggi et al., 2023). Autopsy studies of PD patients with RBD have revealed significant α-synuclein deposits in the subcortical and brainstem nuclei, suggesting that the pathogenesis of RBD may involve regions such as the ventral-lateral gray matter around the aqueduct, the lateral pontine tegmentum, and the nucleus of the pontine pedunculi (Lu et al., 2006; Luppi et al., 2011). Central autonomic network (CAN), which controls autonomic function, is located in brainstem areas such as the periaqueductal gray matter of the midbrain and the parabrachial nucleus of the pons. α-syn deposition in the CAN region is observed in the early stages of PD, which in turn affects autonomic function in PD patients. There is an anatomical overlap between the cranial lesion area of RBD and CAN, which may be the reason for the co-morbidity of the two (Ashraf-ganjouei et al., 2021). Recently, a study by Eckhardt et al. (2023) further demonstrated that PD patients who experiencing autonomic dysfunction share common lesion areas with sleep disorders. Gastrointestinal and cardiovascular dysfunctions in PD are thought to result from degenerative changes in neurons near the brainstem, which also contribute to associated sleep disorders. Additionally, orthostatic hypotension (OH) has been identified as a predictor of REM sleep without atonia (RWA), and the coexistence of these symptoms may indicate a more advanced stage of PD (Eckhardt et al., 2023).

#### 3.2.3 Relationship to emotions

Anxiety and depression are important non-motor symptom of PD. In recent years, many studies have explored the relationship between autonomic dysfunction and mood disorders in PD patients. In Sklerov et al. (2020) conducted a longitudinal study of PD patients with autonomic dysfunction over a period of 60 months. They found that autonomic dysfunction worsened, the patients became progressively more depressed, which further influence their daily lives, particularly in the early stages of PD (Sklerov et al., 2020). In line with other studies, the relationships between autonomic dysfunction and mood disorders such as anxiety and depression in PD may be explained by the overlap of the neural substrates involved in both. Key regions of the central autonomic network (e.g., hypothalamus, anterior cingulate cortex, amygdala, insula) are involved in regulating the balance between sympathetic and parasympathetic activity and also play an important role in mood regulation. Some of these regions are also vulnerable to the accumulation of α-synuclein in PD. Dysregulation of neurotransmitter systems, including norepinephrine and epinephrine, has been linked to both mood and autonomic symptoms (Sklerov et al., 2022). Dysbiosis of gut microbiota in gastrointestinal autonomic dysfunction can lead to the release of lipopolysaccharides, which may ascend along the vagus nerve, circumvent the blood-brain barrier, and excessively activate the hypothalamic-pituitary-adrenal (HPA) axis, resulting in anxiety-like behaviors (Chan et al., 2022). Based on these findings, Sklerov et al. (2020) proposed that treating depression in these individuals may be more effective using drugs that block norepinephrine reuptake. However, they emphasized that further research is needed to determine the correlation between the use of antidepressant medications and the ability to improve autonomic dysfunction and how this might affect daily life for PD.

Although several studies have shown that PD autonomic dysfunction is correlated with anxiety and depression, most do not mention which specific type of autonomic dysfunction is correlated with anxiety or depression. In contrast, Adrianna M and others found that gastrointestinal functioning has a stronger correlation with anxiety and depression than other system dysfunctions through a 5-year follow-up. They hypothesized that the relationship between anxiety and depression and gastrointestinal dysfunction could be related to gut-brain axis interactions. Pathologic processes such as intestinal flora imbalance and inflammation may increase the risk of anxiety and depression in patients with PD by promoting cytokine production, disrupting the blood-brain barrier, and causing inflammation or neuronal dysfunction in the central nervous system. However, it is also suggested that anxiety and depression may themselves lead to changes in the gastrointestinal tract, including alterations in the microbiome composition. In addition, thermoregulatory dysfunction is a unique predictor of anxiety and depression, while urinary and cardiovascular dysfunction are primarily associated with depression in PD patients. The follow-up study also observed a trend of worsening depression and anxiety in patients with new-onset PD, which seemed to correlate with the severity of autonomic symptoms. Therefore, it was hypothesized that interventions and treatments for autonomic symptoms in the early stages of Parkinson’s disease may influence the long-term development of emotional symptoms. They argue that future research should explore how autonomic dysfunction interacts with other PD symptoms to influence the trajectory of mood disorders and whether addressing autonomic dysfunction can improve mood (Ratajska et al., 2023).

#### 3.2.4 Relationship to olfactory dysfunction

Both olfactory dysfunction and AutD are among the earliest pre-motor symptoms of Parkinson’s disease (Yoon et al., 2024). A research team from the United States has discovered that olfactory dysfunction is related to gastrointestinal dysfunction, cardiovascular dysfunction, and pupillary motor function in PD (Kang et al., 2012). Although previous studies have maintained that olfactory dysfunction in PD is associated with both sympathetic and parasympathetic nerve dysfunctions of the heart, the team of this research contends that olfactory dysfunction is primarily positively correlated with parasympathetic nerve dysfunctions, while having a relatively minor connection with sympathetic nerve dysfunctions (Goldstein and Sewell, 2009; Oka et al., 2010; Kang et al., 2012).

Recently, the “Revised Single-Hit Hypothesis” posits that PD might commence in the enteric nervous system or the olfactory bulb and exhibit distinct clinical manifestations based on the different sites of onset (Borghammer et al., 2022). Furthermore, PD can be categorized into two disparate subtypes according to the distinct transmission pathways of α-syn: (1) Body-first PD: α-syn accumulates in the peripheral nervous system or the enteric nervous system, and subsequently diffuses along the vagus nerve before ultimately invading the central nervous system. (2) Brain-first PD: α-syn initiates secondary diffusion from the olfactory bulb or amygdala to the peripheral nervous system (Borghammer et al., 2019; Borghammer et al., 2021; Horsager and Borghammer, 2024). As the enteric nervous system is primarily involved in the onset of body-first PD, patients may exhibit autonomic dysfunction as a prodromal symptom.

Yoon et al. (2024) contend that patients with body-first PD would manifest more severe olfactory dysfunction compared to those with brain-first PD. Furthermore, a 7-year follow-up study verified that a greater AutD at the time of diagnosis typically implies a more significant olfactory dysfunction (Stewart et al., 2023). The most recent autopsy studies have proved that, contrary to the common perception, damage to the olfactory bulb does not herald association with olfactory dysfunction; rather, Lewy pathology in the brain is related to olfactory dysfunction (Nag et al., 2019). Hence, Yoon et al. (2024) put forward that the majority of olfactory tests not only demand intact olfactory bulb function but also higher-order cortical function for the correct naming of odors. Moreover, patients with Body-first PD, represented by AutD, have more extensive Lewy body lesions in the new-onset stage and can give rise to higher-order cortical dysfunction, thereby resulting in olfactory dysfunction (Yoon et al., 2024). Secondly, the current hypothesis suggests that due to the distinct transmission pathways of α-syn, in brain-first PD that has its onset in the olfactory bulb, α-syn spreads unilaterally and does not involve both olfactory bulbs, thereby not giving rise to severe olfactory dysfunction (Borghammer, 2021). Nevertheless, in body-first PD where AutD predominates, when α-syn retrogradely spreads through the bilateral vagus nerves to reach the locus coeruleus, it can be projected from the locus coeruleus to both olfactory bulbs, and the simultaneous impairment of both olfactory bulbs results in more pronounced olfactory dysfunction (Kebschull et al., 2016; Stewart et al., 2023).

## 4 Treatment

A new review from the Movements Disorders Society Evidence-Based Medicine provides a list of drugs that could be effective for each of these systems (Seppi et al., 2019), and the drugs that may be effective for the cardiovascular system are droxidopa, fludrocortisone, midodrine, and domperidone (Joseph et al., 1993; Schoffer et al., 2007; Hauser et al., 2014a; Hauser et al., 2014b; Smith et al., 2016; Schreglmann et al., 2017). Domperidone should be used with caution in PD patients with heart disease. Solifenacin is the only drug thought to be potentially effective for urinary symptoms (Zesiewicz et al., 2015). For patients with constipation, Polyethylene glycol, Lubiprostone, Probiotic strains and prebiotic fibers may have desirable effects (Zangaglia et al., 2007; Ondo et al., 2012; Barichella et al., 2016; Leta et al., 2021b). No ideal drug is given for thermoregulatory disorders. Sildenafil, on the other hand, is recommended for male sexual dysfunction (Bernard et al., 2016; Seppi et al., 2019). Fipamezole, which primarily antagonizes the α2-adrenergic receptor and has a moderate affinity for the 5 -hydroxytryptamine transporter and histamine receptor and a weak affinity for other receptors and transporters, was initially investigated as an antimotor disorder drug (Leta et al., 2019), with one of the adverse effects being an elevation in blood pressure, and was therefore preliminarily investigated in the NCT00758849 clinical trial to see if it could be used to treat OH, but the results have not yet been published (Rukavina et al., 2022). Tomoxetine, a norepinephrine transporter protein blocker that delays postsynaptic reuptake of released norepinephrine, is thought to be applicable and potentially efficacious in PD patients with OH (Shibao et al., 2007; Ramirez et al., 2014; Palma and Kaufmann, 2018). Pyridostigmine can act by increasing cholinergic tone in sympathetic ganglia, and was found to be effective in an open-label study of PD patients treated with Pyridostigmine alone. Patients with OH treated with Pyridostigmine had better results than midodrine alone or a combination of the two (Byun et al., 2017).

Untreated constipation in PD can interferes with the absorption of oral levodopa in the small intestine and can lead to life-threatening complications such as sigmoid colon torsion and bowel perforation. Given that dysregulation of intestinal flora is now believed to be a pathogenetic factor in gastrointestinal dysfunction in PD, approaches targeting intestinal flora modulation—such as probiotics and fecal microbiota transplantation (FMT)—are considered potentially effective (Metta et al., 2021). Linaclotide and Prucalopride have been shown to be effective in small studies, but further randomized controlled trials are needed (Freitas et al., 2018). Elobixibat, which increases bile acid concentrations in the colon, stimulates colonic transit and secretion, and is effective in treating constipation in the general population, is also thought to be beneficial for PD patients (Nakajima et al., 2018; Rukavina et al., 2022). A recent study by a team from China also found that acupuncture can be used as an adjunctive treatment for constipation without causing side effects, but further research is needed to determine its long-term effectiveness and safety (Zhang et al., 2023).

Mirabregon, a selective β3-adrenoceptor agonist that induces relaxation of the urethral muscle, has been shown to be useful in the treatment of patients with PD and has an acceptable incidence of adverse events (Peyronnet et al., 2018; Cho et al., 2021). Additionally, transvesical detrusor injections of botulinum toxin A may be effective in PD patients who have not responded to other treatments (Kulaksizoglu and Parman, 2010; Giannantoni et al., 2011; Quarracino et al., 2020).

A recent study of PD patients after deep brain stimulation (DBS) found some improvement in temperature perception, as well as reductions in hyperhidrosis and heat intolerance. However, due to the small sample size and short follow-up period, further research is needed to validate these findings (Leta et al., 2021a; Zhang et al., 2022).

## 5 Conclusion

Autonomic dysfunction is a common non-motor symptom of Parkinson’s disease. It seriously affects patients’ quality of life and has the potential to exacerbate their motor deficits, thereby both the care and financial burden on PD patients (Pfeiffer, 2020; Bloem et al., 2021). While research into autonomic dysfunction is gaining traction, its exact pathogenesis remains unclear and requires further investigation. Autonomic dysfunction is likely to co-exist with motor and other non-motor symptoms of PD, and some may share common pathogenetic mechanisms. Therefore, it is crucial to determine whether there is a causal relationship or merely a correlation, whether multiple symptoms can be treated simultaneously, and whether improving autonomic dysfunction can alleviate other comorbidities. The treatment and management of autonomic dysfunction in patients with PD is very challenging. It may be overlooked by the patient in the early stages or interfered with by other conditions present in the patient. Therefore, clinicians should carefully identify and select potentially useful medications. Future research should focus on exploring potential new treatments for autonomic dysfunction as they are developed (Quarracino et al., 2020).
